# Atypical splicing variants in *PKD1* explain most undiagnosed typical familial ADPKD

**DOI:** 10.1038/s41525-023-00362-z

**Published:** 2023-07-07

**Authors:** Yvonne Hort, Patricia Sullivan, Laura Wedd, Lindsay Fowles, Igor Stevanovski, Ira Deveson, Cas Simons, Andrew Mallett, Chirag Patel, Timothy Furlong, Mark J. Cowley, John Shine, Amali Mallawaarachchi

**Affiliations:** 1 Molecular Genetics of Inherited Kidney Disorders Laboratory, Garvan Institute of Medical Research, Sydney, Australia; 2 Children’s Cancer Institute, Lowy Cancer Centre, UNSW Sydney, Kensington, NSW Australia; 3 School of Clinical Medicine, UNSW Medicine & Health, UNSW Sydney, Kensington, NSW Australia; 4 Centre for Population Genomics, Garvan Institute of Medical Research and UNSW Sydney, Sydney, NSW Australia; 5 Genetic Health Queensland, Royal Brisbane and Women’s Hospital, Herston, QLD Australia; 6 Genomic Technologies, Garvan Institute of Medical Research, Sydney, Australia; 7 Centre for Population Genomics, Garvan Institute of Medical Research and Murdoch Children’s Research Institute, Sydney, Australia; 8 Centre for Population Genomics, Murdoch Children’s Research Institute, Melbourne, VIC Australia; 9 Department of Renal Medicine, Townsville University Hospital, Townsville, QLD Australia; 10 Institute for Molecular Bioscience, The University of Queensland, Brisbane, QLD Australia; 11 College of Medicine and Dentistry, James Cook University, Townsville, QLD Australia; 12 Clinical Genetics Service, Institute of Precision Medicine and Bioinformatics, Royal Prince Alfred Hospital, Sydney, Australia

**Keywords:** Polycystic kidney disease, Next-generation sequencing, Genetic testing, Medical genomics, RNA splicing

## Abstract

Autosomal dominant polycystic kidney disease (ADPKD) is the most common monogenic cause of kidney failure and is primarily associated with *PKD1* or *PKD2*. Approximately 10% of patients remain undiagnosed after standard genetic testing. We aimed to utilise short and long-read genome sequencing and RNA studies to investigate undiagnosed families. Patients with typical ADPKD phenotype and undiagnosed after genetic diagnostics were recruited. Probands underwent short-read genome sequencing, *PKD1* and *PKD2* coding and non-coding analyses and then genome-wide analysis. Targeted RNA studies investigated variants suspected to impact splicing. Those undiagnosed then underwent Oxford Nanopore Technologies long-read genome sequencing. From over 172 probands, 9 met inclusion criteria and consented. A genetic diagnosis was made in 8 of 9 (89%) families undiagnosed on prior genetic testing. Six had variants impacting splicing, five in non-coding regions of *PKD1*. Short-read genome sequencing identified novel branchpoint, AG-exclusion zone and missense variants generating cryptic splice sites and a deletion causing critical intron shortening. Long-read sequencing confirmed the diagnosis in one family. Most undiagnosed families with typical ADPKD have splice-impacting variants in *PKD1*. We describe a pragmatic method for diagnostic laboratories to assess *PKD1* and *PKD2* non-coding regions and validate suspected splicing variants through targeted RNA studies.

## Introduction

Autosomal dominant polycystic kidney disease (ADPKD) is the most common monogenic cause of kidney failure, affecting approximately 1 in 1000 people^1^. The condition is primarily caused by disease-causing variants in *PKD1* and *PKD2*. Genetic diagnosis of ADPKD is technically challenging due to six pseudogenes that are >97% homologous in sequence to the genuine *PKD1* gene^1^. This sequence homology has driven the development of specific genetic diagnostic techniques to robustly sequence *PKD1* that have focussed mainly on the analysis of the protein-coding regions of *PKD1, PKD2* and then the wider exome. These techniques include long-range polymerase chain reaction (LR-PCR) and Sanger sequencing, targeted next-generation sequencing using probes specific to coding regions of cystic-related genes (tNGS), exome sequencing and genome sequencing with coding-based analysis^1–6^. The diagnostic yield from these studies differs based on the clinical breadth of the cohort, ranging from approximately 60% in phenotypically broad cohorts to >90% in cohorts tightly selected for features typical of *PKD1* and *PKD2*-mediated disease^1,3–5,7^. Even with the most stringent coding-based analysis, at least 7% of ADPKD families are left without a genetic diagnosis^7^.

In recent years, there have been substantial advances in understanding the breadth of ADPKD gained through investigating genetically undiagnosed patients. New genes have been identified that contribute to the ADPKD spectrum, including *GANAB, DNAJB11, IFT140, ALG5, ALG8* and *ALG9*^5,7,8^. Despite these advances in disease knowledge and extensive coding-region focussed analysis, there remains a cohort of patients with a typical ADPKD phenotype who are without a genetic diagnosis. It is thus an open question as to whether this is due to technical limitations in identifying causative variants within *PKD1* or *PKD2* or the existence of an unknown ‘PKD3’ gene that is associated with a typical ADPKD phenotype.

We aimed to address this question by investigating a cohort selected to have a typical ADPKD phenotype, a positive family history and be undiagnosed on standard diagnostic genetic testing. We aimed to investigate whether these patients had variants in previously unidentified genes or, as other diseases suggest, novel variants in the most likely genes of interest – *PKD1* and *PKD2*. To approach this challenge, we applied sequencing methods not previously extensively used in ADPKD, including short and long-read genome sequencing combined with targeted RNA sequencing.

We have previously shown that short-read genome sequencing is a robust diagnostic method in ADPKD, allowing the detection of single nucleotide, short indel and structural variants^1,4^. However, though the whole genome is sequenced, current diagnostic laboratory protocols essentially limit analysis to protein-coding regions of the genome. This particularly biases against the detection of non-coding variants that may impact splicing. To date, there has been limited study of potential atypical splice-impacting variants in ADPKD and how best to predict their pathogenicity^9–11^. Another challenge for diagnostic laboratories is in clarifying the pathogenicity of identified variants of uncertain significance (VUS). Even if non-coding variants are identified, pathogenicity confirmation of these variants typically requires functional analysis that is not routinely performed in diagnostic laboratories^12^. Pathogenicity can also be clarified in some instances by phasing of variants (confirming which allele the variant is present on), which is not usually possible with short-read sequencing. More recently available long-read technologies, such as Oxford Nanopore Technologies (ONT), have been shown to inform phasing in other disease groups, but this has not been previously applied in ADPKD^13^. Our previous studies in genome sequencing diagnostics in ADPKD have informed this study, and patients left undiagnosed from these previous cohorts were assessed for suitability for this study^1,4^. In this study, we report for the first time the combination of short and long-read genome sequencing with whole genome analysis and RNA studies to investigate ADPKD families without a diagnosis after standard diagnostic genetic testing.

## Results

**Fig. 1:**
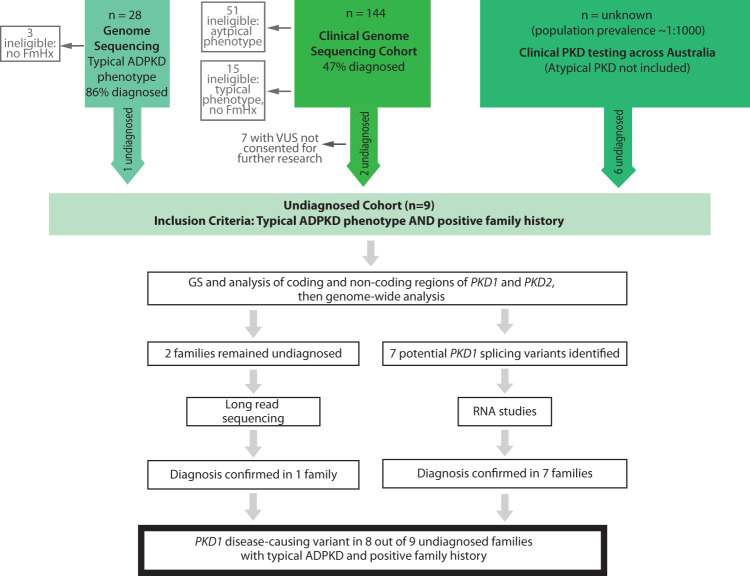
Study design. Overview of patients assessed for study suitability and study method. VUS variant of uncertain significance, GS genome sequencing; FmHx family history.

Over 172 patients were assessed for suitability for recruitment. This included 28 patients from a cohort of typical ADPKD who had undergone genome sequencing, 144 patients from a cohort of suspected ADPKD who had undergone diagnostic genome sequencing and a cohort of patients with typical and atypical PKD reviewed at multidisciplinary kidney genetics clinics from across Australia who had undergone diagnostic genetic testing^1,4,14^. From this initial pool of over 172 probands, 9 families were recruited who met the study inclusion criteria (Fig. 1). Patients with atypical clinical features or no family history were deemed ineligible as they did not meet the inclusion criteria. Recruitment was restricted to those with a family history of ADPKD in order to target analysis toward inherited germline rather than mosaic variants. All patients had previously undergone standard diagnostic genetic testing via LR-PCR of *PKD1* and *PKD2* coding regions and massively parallel sequencing of this PCR-product (2 probands) or diagnostic genome sequencing with analysis targeted to coding regions of a cystic kidney disease gene panel (6 probands) or both (1 participant) (Supplementary Table 2). An additional seven patients met the inclusion criteria and had a VUS identified in *PKD1* on initial diagnostic genetic testing but were not consented to further research analysis and therefore did not proceed to this study (Fig. 1).

Four of nine probands had ESKD (End Stage Kidney Disease), and all had enlarged kidney lengths with numerous kidney cysts on imaging (Table 1 & Supplementary Table 2). Five of nine probands had extra-renal features of ADPKD reported (Supplementary Table 2).

**Table 1:** Patient characteristics and overall results.

Family ID	Patient ID	Kidney function^a^/age (yrs)^b^	Extra-renal features	Gene	c.^c^	p.^d^	Variant type	Variant classification^e^	Additional studies performed
FRBW403	RBW403	>90/31	Y	*PKD1*	c.11017-25 A > G	p.?	Splice variant - branchpoint variant	Likely Pathogenic (PM2; PS3_strong)	WGS; RNA studies
FRPA028	RPA028	>90/23	N	*PKD1*	c.11017-25 A > C	p.?	Splice variant - branchpoint variant	Likely Pathogenic (PM2; PS3_strong)	WGS; RNA studies
FRPA014	RPA014	ESKD/47	Y	*PKD1*	c.11017-10 C > A	p.?	Splice variant – AG-Exclusion Zone variant	Likely Pathogenic (PM2; PS3_strong, PP5)	WGS; RNA studies
FRPA019	RPA019	>90/35	N	*PKD1*	c.1991C>T	p.Ala664_Ser699del	Splice variant - generation of new cryptic donor site	Likely Pathogenic (PM2; PS3_strong; PP1)	WGS; RNA studies
	RPA020	44/69	NR						
F19F00138	RBW401	>90/30	NR	*PKD1*	c.10167+25_10167+43del	p.?	Splice variant - intronic deletion causing critical intron shortening	Likely Pathogenic (PS3_strong; PP1; PP5)	WGS; RNA studies
	19F00138	>90/25	NR						
FRPA021	RPA021	ESKD/52	Y	*PKD1*	c.7489+5 G > A	p.?	Splice variant – donor splice site variant	Likely Pathogenic (PM2; PS3_strong)	WGS; RNA studies
RG_0044	RG_0044.0048	ESKD/58	N	*PKD1*	c.2878 G > A	p.(Gly960Ser)	Missense variant	Likely Pathogenic (PP1_moderate; PS4_moderate; PP3; PM5_supporting)	WGS
FRPA017	RPA017	41/38		*PKD1*	c.10118 C > A	p.(Ser3373*)	Nonsense variant	Pathogenic (PVS1; PM2; PS2)	WGS; Long-read sequencing
	RPA015	50/71		*PKD2*	c.1249 C > T	p.(Arg417*)	Nonsense variant	Pathogenic (PM2; PVS1; PP5)	
FRPA007	RPA007	ESKD/45	Y	*PKD1*	c.8471 A > G	p.(Gln2824Arg)	Missense variant	VUS (PM2)	WGS; Long-read sequencing

^a^Estimated GFR as per CKD-EPI (ml/min/1.73 m^2^);

^b^Age kidney function recorded.

^c^*PKD1* NM_001009944.3, *PKD2* NM_000297.4.

^d^*PKD1* NP_001009944.3 *PKD2* NP_000288.1; *ESKD* end-stage kidney disease; *NR* not reported; *VUS* variant of uncertain significance.

^e^ACMG criteria used to arrive at Variant classification included in brackets^17,18^; *WGS* short read whole genome sequencing.

**Fig. 2:**
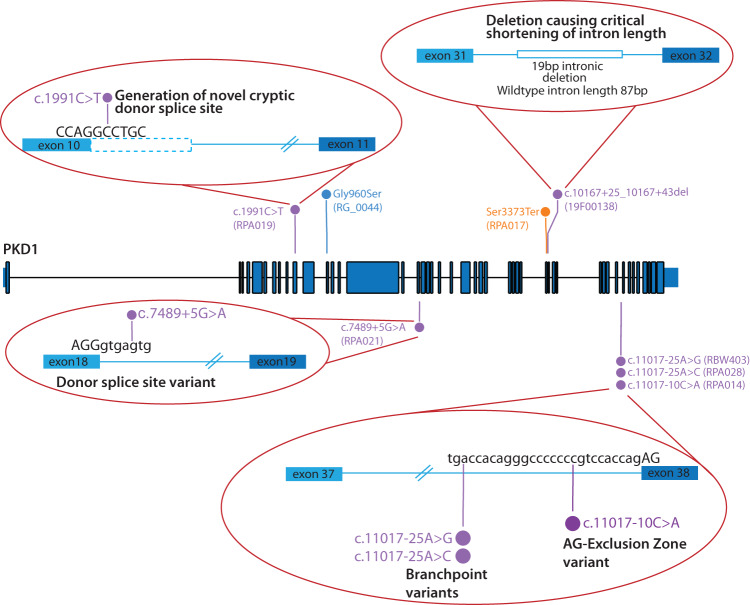
Overview of variants types identified in *PKD1*. Variants identified in the *PKD1* gene in the study, including a range of different splicing variants. Gene illustration developed using Protein Paint^41^. *PKD1* NM_001009944.3.

After genome sequencing and whole genome analysis, a genetic diagnosis (identification of a Pathogenic or Likely Pathogenic variant) was made in eight out of nine families, with all having disease-causing variants in *PKD1* (Table 1 and Fig. 2). An additional family (FRPA007) had a VUS identified in *PKD1*. Six of the disease-causing variants were shown through RNA studies to impact splicing. Four of these splicing variants had been identified on the initial diagnostic testing (including one coding variant) but classified as of uncertain significance, with segregation studies not able to clarify pathogenicity (Supplementary Table 2).

### *PKD1* intron 37 splice-impacting variants

**Fig. 3:**
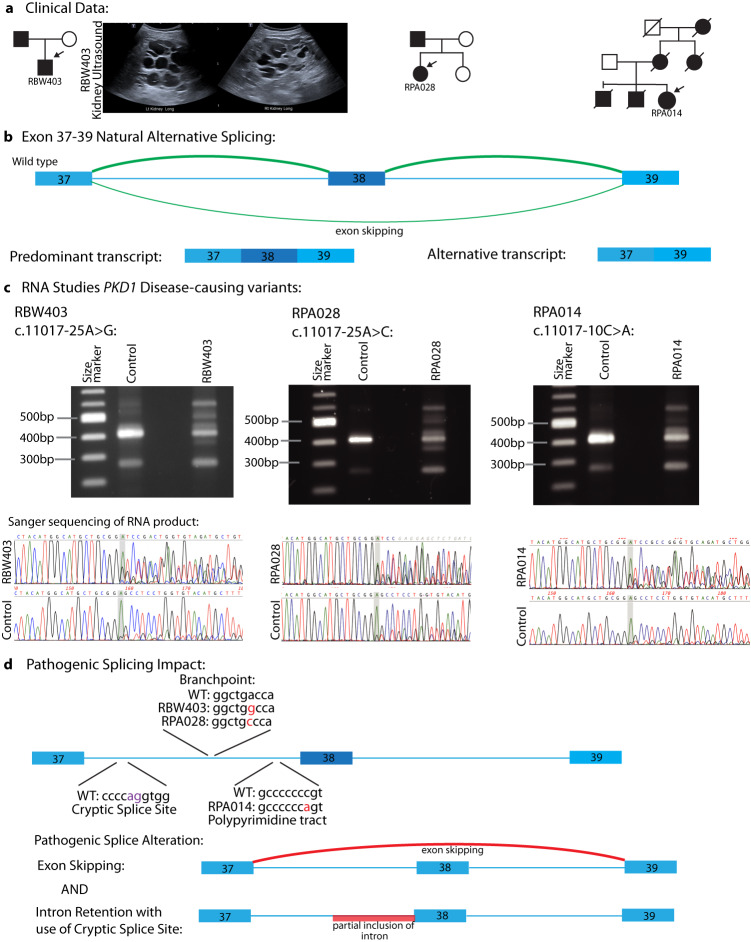
Disease-causing splicing variants in *PKD1* intron 37. **A** Pedigrees and renal ultrasound images from RBW403 demonstrating bilateral kidney cysts. **B** Natural splicing of exons 37, 38, and 39 of *PKD1*, depicting that skipping of exon 38 naturally occurs at a low level. **C** RT-PCR studies and Sanger sequencing of RT-PCR product in RBW403, RPA028, and RPA014. Additional bands are demonstrated in the affected individuals compared with controls, consistent in size with skipping of exon 38 and partial retention of exon 37. This is also reflected in Sanger sequencing of the RT-PCR product. Low-level skipping of exon 38 is evident in the controls. **D** Illustration of the splicing impact of the different variants identified in exon 37 across the cohort. All three variants result in the skipping of exon 38 and, less frequently, partial retention of exon 37 due to the use of an upstream cryptic splice site.

Patient RBW403 had a clinical diagnosis of ADPKD made at 12 years of age in the context of a known diagnosis in his father, who reached ESKD at 44yo (Table 1 and Fig. 3A). Through this study, a novel variant was identified in intron 37 of *PKD1* (c.11017-25 A > G) that was predicted by in silico splice prediction tool, introme, to interrupt the splicing branchpoint (Fig. 3C, D). This variant was absent in control databases and not previously reported in ADPKD cohorts. This variant had been detected on both previously performed diagnostic tests (next-generation sequencing of LR-PCR amplicons targeted to *PKD1* and *PKD2* and then genome sequencing) but predicted benign based on available in silico tools. Introme predicted multiple potential splicing impacts. The predominant interpretation was that this branchpoint variant would largely result in the skipping of exon 38, introducing a premature stop codon. An alternate interpretation was that the presence of a wildtype cryptic splice site 156 base pairs upstream of the c.11017-25 A > G branchpoint variant would result in retention of 180 bp of intron 37 at a reduced frequency (Fig. 3D). RNA studies revealed evidence for both splicing outcomes, with the skipping of exon 38 being far more prevalent than intron retention. A review of control GTEx RNA data suggests low-level (5%) natural alternative splicing of exon 38 in kidney samples (Fig. 3B and Supplementary Table 3).

Two additional patients in this cohort (RPA028 and RPA014, Table 1 and Fig. 3A) were identified to have different variants in intron 37 that were also predicted to interrupt the usual function of the exon 38 acceptor splice site (Fig. 3C, D). RPA028 had a different nucleotide substitution (*PKD1* c.11017-25 A > C), interrupting the same branchpoint as in RBW403. RPA014 had a variant 10 base pairs from the start of exon 38 (*PKD1* c.11017-10 C > A) that impacted the acceptor splice site through the inclusion of the ‘AG’ dinucleotide in the AG Exclusion Zone^15^. RNA studies in both patients demonstrated a similar impact to that seen in RBW403, with a combination of skipping of exon 38 and partial retention of intron 37 (Fig. 3C).

### Generation of novel cryptic donor splice site

Patient RPA019 had a clinical diagnosis of ADPKD made at 29yo during screening as a potential kidney donor for her affected brother (Table 1 and Supplementary Fig. 1A). Previous diagnostic testing had identified a missense variant classified as a VUS in exon 10 of *PKD1* (c.1991 C > T). RPA019 underwent genome sequencing and whole genome analysis, and no additional phenotype-relevant variants were identified. Introme predicted that the c.1991C > T variant would generate a new cryptic donor site and result in an in-frame deletion of 36 amino acids (Supplementary Fig. 1C). RNA studies supported this prediction (Supplementary Fig. 1B). The variant was segregated to the proband’s affected mother and was absent in population datasets, though alternate amino acid substitutions at the same residue and substitutions at the same nucleotide are reported in population datasets. To our knowledge, this variant has not previously been reported in ADPKD cohorts.

### Intronic deletion causing critical shortening of intron length

19F00138 and her sister (RBW401) both had a clinical diagnosis of ADPKD, with bilateral kidney enlargement, multiple kidney cysts and multi-generational family history of ADPKD (Supplementary Fig. 1D). Clinical short-read genome sequencing in 19F00138 had been non-diagnostic. Re-analysis of the genome sequencing data identified a 19 bp deletion within intron 31 of *PKD1* that was predicted to result in shortening of the intron beneath its critical length and, therefore, intron retention^16^ (Supplementary Fig. 1E). This variant was segregated to RBW401 and RNA studies in her demonstrated retention of intron 31, creating a frameshifting insertion (Supplementary Fig. 1F). This variant has been reported previously in a patient with a de novo ADPKD phenotype^17^.

### Extended donor splice site variant

RPA021 and her brother both had a clinical diagnosis of ADPKD, with both undergoing kidney transplantation in their 50s (Supplementary Fig. 2A, B). In our previous study^1^, a variant of uncertain significance had been identified in intron 18 of *PKD1* (c.7489+5 G > A). Though suspicious for being disease-causing via disruption of the native splice site, there was insufficient evidence to confirm pathogenicity without support from functional studies. This variant has also been reported previously by our research group in an unrelated patient who was part of a cohort of patients who underwent clinical PKD testing via short-read genome sequencing^4^. The patient in this previous study (Pt D158) was not known to be related to FRPA021 and did not share ethnicity^4^. RNA studies in RPA021 demonstrated that the c.7489+5 G > A variant in *PKD1* resulted in the retention of 93 base pairs of intron 18, introducing a premature stop codon (Supplementary Fig. 2C, D).

### Coding *PKD1* variants

The RG_0044 family had a multi-generational history of ADPKD (Supplementary Fig. 3). Participant RG_0044.0048 had previously undergone diagnostic genetic testing via next-generation sequencing of LR-PCR amplicons targeted to *PKD1* and *PKD2*, and no clinically significant variants had been identified. Genome sequencing and analysis identified a previously reported, likely pathogenic missense variant in *PKD1* p.(Gly960Ser*)* that had not been identified on the previous diagnostic testing^18^. This variant was appropriately segregated to six affected and unaffected family members (Supplementary Fig. 3).

**Fig. 4:**
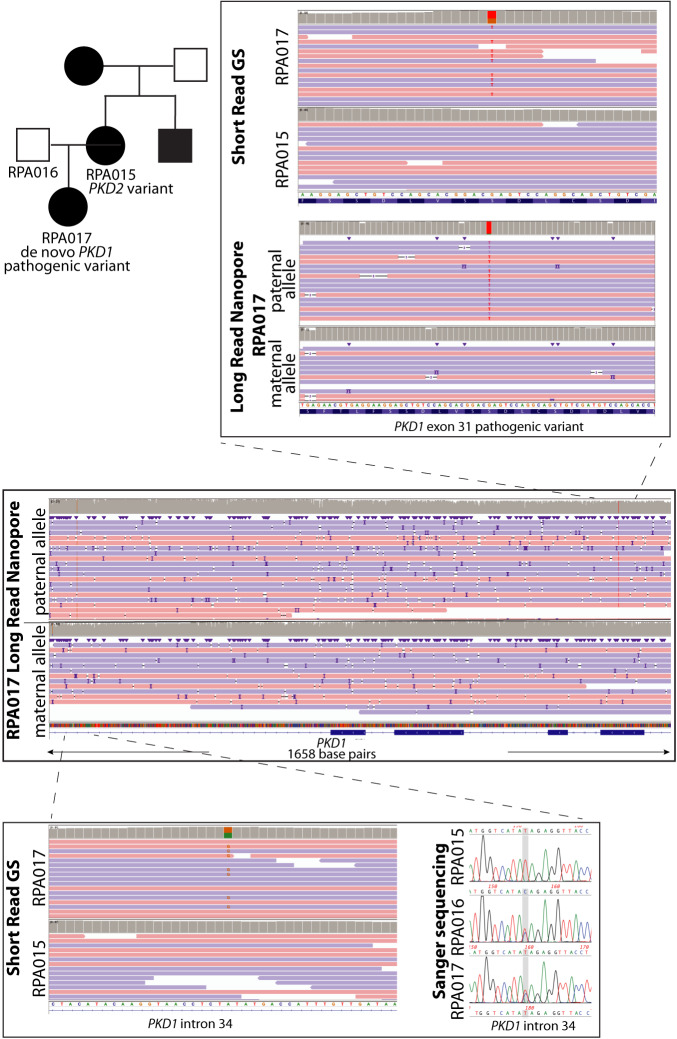
Long read nanopore sequencing confirms de novo *PKD1* variant occurred on the paternal allele. The middle panel shows long-read genome sequencing data from RPA017 over *PKD1* exons 31–35, separated into maternal and paternal alleles. The top panel ‘zooms in’ over the region that includes the pathogenic *PKD1* exon 31 variant identified in RPA017. The variant is absent in RPA015 (affected mother). The bottom panel ‘zooms in’ over an intronic single nucleotide variant identified in RPA017 that short-read GS and Sanger sequencing shows is present in RPA016 (unaffected father) and absent in RPA015 (affected mother). In RPA017, this variant is seen on the same long-read sequencing as the pathogenic *PKD1* variant, demonstrating that the de novo disease-causing variant in RPA017 has occurred on her paternally inherited *PKD1* allele. GS genome sequencing.

RPA017 had a clinical diagnosis of ADPKD made at 34yo (Fig. 4 and Supplementary Table 2). She was motivated for a genetic diagnosis to inform IVF and PGD. Her mother (RPA015) was diagnosed with ADPKD with CKD 3a in her 50s in the context of a diagnosis in her mother. RPA017’s father (RPA016) had normal kidney ultrasound at 72yo. Genome sequencing in RPA017 identified a nonsense variant in exon 31 of *PKD1*. However, segregation by LR-PCR and Sanger sequencing demonstrated that this variant was absent in her affected mother and unaffected father (paternity confirmed). Subsequent genome sequencing in RPA015 identified a nonsense variant in exon 5 of *PKD2* that was absent in her affected daughter. Both variants met ACMG criteria for likely pathogenic, though the inheritance pattern in the family was unclear. In addition, PGD for *PKD1* variants is contingent on multigenerational linkage studies. The information from short-read sequencing was not adequate to inform these linkage studies as the parent of origin of the *PKD1* pathogenic allele was unknown. To clarify the inheritance in this family, ONT long-read sequencing was performed to facilitate variant phasing, which demonstrated that the *PKD1* variant identified in RPA017 was present on the allele she inherited from her father. Sanger sequencing showed that the variant was absent in her father’s peripheral blood DNA, strongly suggesting this was a de novo variant in RPA017(Fig. 4). Phasing also confirmed that RPA017 had not inherited the affected *PKD2* allele from her mother. This information was used to inform linkage studies for PGD.

### Undiagnosed

Research analysis performed in RPA007 identified a VUS, p.(Gln2824Arg) in *PKD1* that segregated to her affected mother. Whole genome analysis in RPA007 with both short and long-read genome sequencing did not identify any additional variants of interest. Copy number variant analysis was uninformative. No splicing impact was predicted by introme. The *PKD1* p.(Gln2824Arg) variant is absent in population databases, predicted pathogenic by in silico tools and has not been previously reported in PKD cohorts. This information alone is insufficient to clarify the pathogenicity of this variant.

## Discussion

Pathogenic variants in *PKD1* and *PKD2* have been shown to be responsible for disease in most patients with a typical phenotype of ADPKD. However, genetic sequencing in ADPKD cohorts consistently results in approximately 10% of patients being left without a genetic diagnosis^4,5,7^. This study shows that most of these undiagnosed families have splice-impacting variants in *PKD1* that were uncertain or undetected on standard diagnostic genetic testing. We demonstrate the value of sequencing and, importantly, analysis of the protein-coding and non-coding regions of *PKD1* and *PKD2*, combined with targeted RNA studies to confirm a genetic diagnosis. For the first time, we show the value of long-read sequencing in ADPKD to inform the phasing and inheritance of variants.

Utilising a genetic diagnosis to inform clinical care requires a definitive genetic result. Cascade testing can only be offered in families with a definitive genetic diagnosis, and this is the same for using genetic results to inform family planning. This highlights the value of improving diagnostic yield for families with ADPKD and the value of the results of this study, which demonstrates that a significant proportion of undiagnosed families have variants that affect gene splicing. There is increasing evidence for similar variants across other disease groups, where, for example, RNA-sequencing in a cohort of patients with undiagnosed muscle disease identified a diagnosis through aberrant splicing in 35% of patients^19^. RNA-sequencing often requires analysis of a tissue of interest rather than using blood RNA^12,19,20^. In kidney disease, RNA extracted from kidney tissue or urothelial cells is technically more challenging to access^12^. We demonstrate a practical approach for evaluating suspected splicing variants in ADPKD using RT-PCR of total RNA extracted from peripheral blood. This pragmatic, targeted approach provides functional evidence to classify VUS that results in substantial splicing defects without requiring access to kidney tissue and is achievable for a diagnostic laboratory to replicate^21^. Developing protocols for diagnostic genetic laboratories to identify and then confirm coding or non-coding aberrant splicing variants is key in improving current genomic diagnostic rates^12^. The high diagnostic yield in our study highlights the importance of diagnostic laboratories analysing beyond the coding region in patients with a typical ADPKD phenotype by using robust in silico tools, such as Introme^22^. Importantly, we also show that RT-PCR can then be used to evaluate potential splicing variants identified through this broader analysis. The homologous *PKD1*-pseudogenes produce mRNA transcripts that are approximately 97% homologous to the 5′ regions of *PKD1* mRNA; therefore, this RT-PCR method requires the use of unique primers to avoid inadvertently amplifying transcripts from the PKD1-pseudogenes^23^.

A confirmed genetic diagnosis is increasingly becoming the standard of care for families with genetic disorders and is being utilised across all inherited kidney diseases, including ADPKD^1,4,24–26^. A genetic diagnosis allows early definitive diagnosis in ADPKD, which can provide prognostic information and allow for early institution of treatments, including vigorous hypertension management and tolvaptan for those predicted to have more rapidly progressive disease^4^. Genetic diagnosis also allows for informing the selection of kidney donors and for family planning. In this cohort alone, several families utilised a confirmed genetic diagnosis to inform PGD of embryos. In many jurisdictions, such as Australia, New Zealand and the United Kingdom, national health service subsidies are available for ADPKD families for IVF and PGD, highlighting that providing genetic counselling to ADPKD families is an essential aspect of their care^27^. ADPKD is one of the most common monogenic conditions screened for in IVF and PGD^28^. There is also increasing evidence of nocturnal hypertension in children with ADPKD, suggesting that guidelines regarding the diagnosis of ADPKD in childhood may be modified in the future to recommend early intervention for these children^29^. Imaging results can be variable in paediatric populations, whereas genetic diagnostics allows reliable, definitive diagnosis^29^^,30^.

Classifying VUS is currently a challenge for diagnostic laboratories, particularly in ADPKD, where variants are often private to families and multiple samples from large pedigrees are typically not available to perform segregation studies to clarify pathogenicity. We also demonstrate the additional value of long-read sequencing in understanding the pathogenicity of variants in ADPKD. Long-read technologies have the additional advantage of allowing the phasing of variants, which has obvious applications in confirming bi-allelic inheritance in autosomal recessive disease. In autosomal dominant disorders, long-read sequencing allows the opportunity to identify the parental allele on which a de novo variant occurs. This is valuable in understanding inheritance in complex families, such as we demonstrate in family FRPA017. Another unique application is in informing linkage studies for couples undergoing PGD for de novo ADPKD. For couples undergoing PGD, detailed phasing studies are performed to identify accurate markers that are then used to ascertain affected vs unaffected embryos. For patients with *PKD1*-mediated disease, this requires samples from multiple generations of the affected family, as direct sequencing of *PKD1* is hampered by the presence of the homologous pseudogenes. This makes PGD challenging to access for patients with de novo *PKD1*-disease, who are the only affected person in their family. In this situation, which impacts 10% of patients with ADPKD, long-read sequencing can provide important phasing information that can allow these patients to access PGD of embryos^31^. Long-read data may additionally be used to detect structural variants that are missed by short-read sequencing, although we did not detect any relevant events here^13^.

In order to maximise the study size, this cohort was collected from a larger pool of over 172 patients from across Australia who had undergone standard diagnostic genetic testing for PKD. Given the high yield of diagnostic testing in ADPKD and the strict inclusion criteria of this study, only 16 patients from this larger pool met the inclusion criteria, of which 9 consented to participation. Inclusion was deliberately limited to patients with a family history of ADPKD to focus on germline rather than mosaic variants, restricting the eligible pool of patients^32^. Our results are comparable to other disease groups and highlight that previously unrecognised or undetected splice variants may be causative in these families^33^. Our smaller cohort size means there is value in applying this method to a larger cohort.

Our results show that for families with a typical ADPKD phenotype, variants are most likely to be found in *PKD1* and *PKD2* rather than other PKD-associated genes and that most of these variants are splice-impacting. We provide evidence of the value of diagnostic laboratories expanding the analysis to non-protein-coding regions to improve diagnostic yield in ADPKD – we achieved this through WGS, though other validated sequencing methodologies could also be utilised. Importantly, we describe an achievable method for assessing uncertain variants that are predicted to impact splicing in this common disorder. ADPKD is the most common inherited kidney disorder and contributes to approximately 10% of kidney failure cohorts^4^. Improving diagnostic rates allows for improved management through earlier institution of treatment and access to holistic care that includes genetic counselling. Importantly, improving the understanding of the underlying genetic basis for all families with ADPKD is a critical step in developing personalised therapies for this common genetic disease.

## Methods

### Enrolment and inclusion criteria

We enrolled patients with typical ADPKD clinical features and a family history of ADPKD who were without a genetic diagnosis after diagnostic sequencing of *PKD1* and *PKD2* or a larger cystic gene panel that included the *PKD1* and *PKD2* genes. Patients undiagnosed from our previous studies were assessed for suitability for this study (Fig. 1). In addition, patients were recruited from clinical sites across Australia. Family members were recruited as required and available. Ethics approval for the study was obtained from the RPAH Human Research Ethics Committee (HREC/18/RPAH/726). All participants provided written informed consent. The authors have received and archived written patient consent. All data included is de-identified.

Clinical, family and imaging data were obtained during the clinical review or review of medical records. Kidney lengths were based on ultrasound measurements as kidney ultrasound is the Medicare-funded imaging modality available for the assessment of ADPKD patients in Australia. The kidney function was calculated using the CKD–EPI equation.

### Short-read genome sequencing

All probands underwent short-read genome sequencing using DNA extracted from peripheral blood samples. Genome sequencing was performed on the HiSeqX sequencing system (Illumina Inc., California, CA, USA) after either PCR-based library preparation (Illumina HiSeq X TruSeq Nano DNA HT Sample Prep Kit) or PCR-free library preparation (KAPA Hyper PCR-free kit, Roche). The sequencing was performed within an ISO17025-accredited laboratory at the Kinghorn Centre for Clinical Genomics within the Garvan Institute. All samples were processed via a custom bioinformatics pipeline based on GATK best practice, which was optimised for the identification of germline variants^1,4^. Reads were aligned to the hg37 reference sequence. Sequence variants were filtered using Seave^34^. CNV and structural variant analysis was performed using ClinSV^35^. Introme was used to assess for variants predicted to impact splicing^22^. Control *PKD1* splice junction usage was obtained using GTEx V8, filtered to include only kidney samples^36^. Initial variant analysis was targeted to coding and intronic and promoter regions of *PKD1* (NM_001009944.3) and *PKD2* (NM_000297.4), with all variants (ranging from predicted high to low impact) manually reviewed. Analysis was then expanded to phenotype-driven whole genome analysis. Variants were classified according to American College of Medical Genetics (ACMG) Guidelines^30,37^. Sanger sequencing (with prior LR-PCR amplification if within the *PKD1*-pseudogene homologous region) was performed to confirm all single nucleotide and short indel variants identified on genome sequencing and for family studies.

### RNA studies

RNA functional studies were performed to assess variants predicted to impact splicing in *PKD1*. Total RNA was extracted from venous blood (Macherey-Nagel Nucleospin RNA Blood Kit) for RT-PCR studies. If the variant of interest was within the PKD1-pseudogene homologous region, amplification was performed with at least one of the primer pairs being unique to the *PKD1* sequence in order to avoid amplifying *PKD1*-pseudogene transcripts (see Supplementary Table 1 for primer sequences). Sanger sequencing was performed on this PCR product. See Supplementary Methods for further details. RNA studies were performed within a research laboratory at the Garvan Institute.

### Long-read sequencing

In families who remained negative after short-read genome sequencing or for whom phasing could inform variant interpretation and classification, long-read sequencing was performed. High molecular weight DNA was sheared to ~20 kb fragment size using Covaris G-tubes. Sequencing libraries were prepared from ~1.5 to 5 µg of sheared DNA using native library prep kits (SQK-LSK110) and sequenced for 72 h on a PromethION (FLO-PRO002, R9.4.1) flow cell. Raw ONT sequencing data was converted to BLOW5 format with slow5tools (v0.3.0)^38^, then base-called using Guppy (4.0.11 or later). Resulting FASTQ files were aligned to the hg38 reference genome using minimap2 (v2.14-r883)^39^, and Longshot (v0.4.1)^40^ was used to identify and phase variants within the *PKD1* locus. Long-read sequencing was performed within a research laboratory at the Garvan Institute.

## Supplementary information

Supplementary Material

Supplementary Table 3: GTex Data

## Data Availability

Variants identified in the study have been submitted to ClinVar (ClinVar Accessions: SCV002756451–SCV002756459). Other data is available upon request. As per the patient’s informed consent, the long-read DNA sequencing data can be made available on request for institutional ethics-approved research groups.
