# Supplementary material for: Atypical splicing variants in PKD1 explain most undiagnosed typical familial ADPKD
Source: NPJ Genom Med. 2023 Jul 7;8:16. doi: 10.1038/s41525-023-00362-z (PMC10328916; doi:10.1038/s41525-023-00362-z)
Supplement: Supplementary file 1 — Supplementary Material [file 41525_2023_362_MOESM1_ESM.pdf]

Supplementary Material

Supplementary Methods

Supplementary Figure 1: Disease-causing variants impacting *PKD1* splicing

Supplementary Figure 2: Splicing studies in FRPA021

Supplementary Figure 3: RG0044 Pedigree

Supplementary Table 1: Primer Sequences and PCR Conditions

Supplementary Table 2: Patient Characteristics and Variant Classification

Supplementary Table 3: GTex Data

Supplementary Methods

Methods for the Targeted RNA studies:

RNA extraction
Total RNA was extracted from peripheral blood using the Machery Nagel Nucleospin RNA Blood Kit following the manufacturer's recommendations.

Reverse Transcription PCR and Sanger Sequencing
Superscript III (Invitrogen) first-strand synthesis system was used to make complementary DNA (cDNA) from 400ng total RNA according to the kit instructions and with the random hexamers included. All samples were also run with a Reverse Transcriptase negative control (RT-).

Amplitaq 360 DNA polymerase (applied biosystems) or LongAmp Taq DNA Polymerase (New England Biolabs) was used for PCR using the primers listed in Supplementary Table 1. Conditions for the PCR varied according to the primer sets, and are included in Supplementary Table 1.

Control cDNA was from healthy individuals, or healthy family members where available. PCR products were analyzed on 1.5 – 1.8% agarose gels. All gels presented derive from the same experiment and were processed in parallel.

PCR products were cleaned up using ExoSAP-IT (affymetrix USB) before Sanger Sequencing. 10-30ng PCR product was sequenced with 3.2pmol sequencing primer. Sanger sequence chromatograms were analysed using MacVector DNA sequence analysis software (MacVector Inc.).

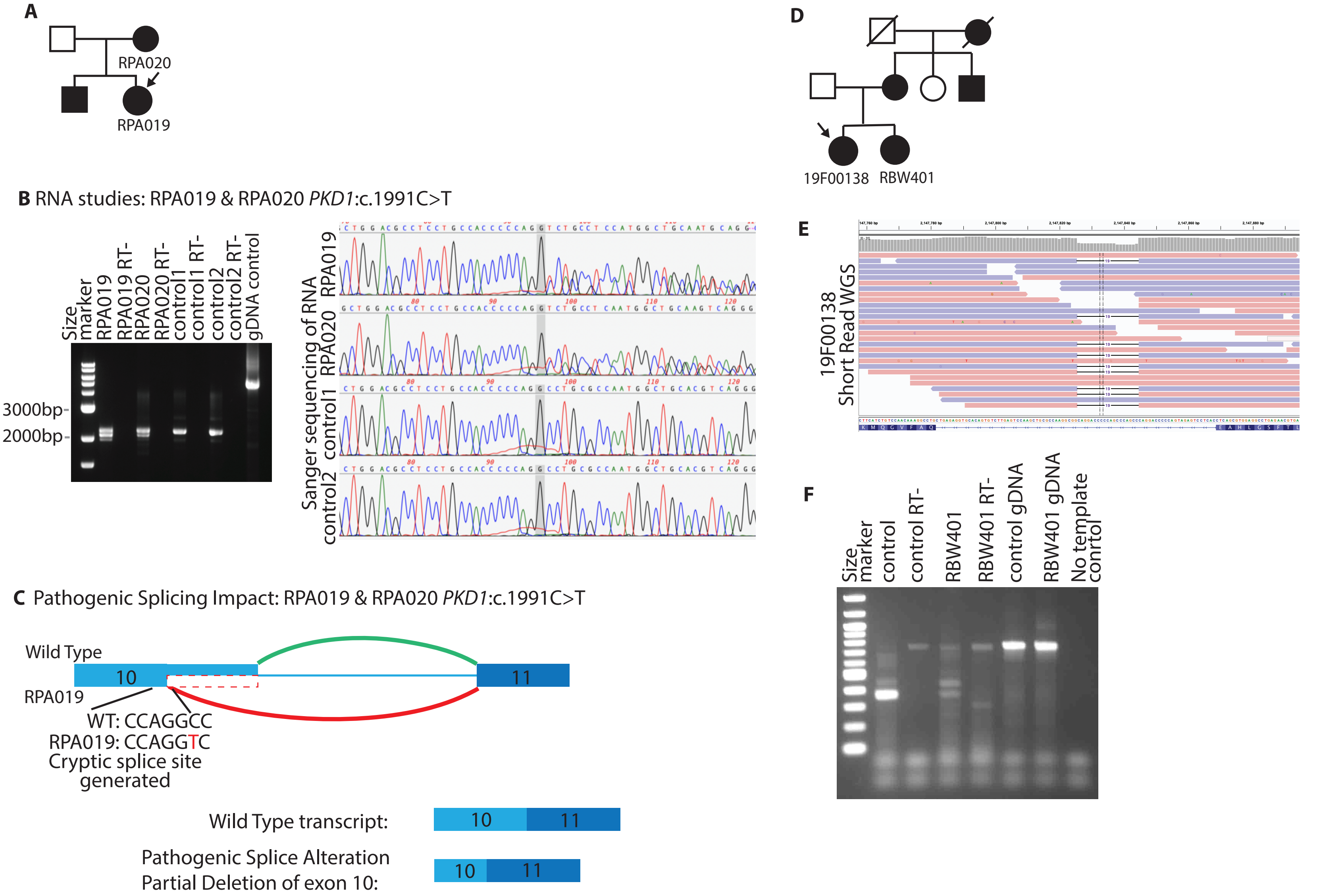

Supplementary Figure 1: Disease-causing variants impacting *PKD1* splicing

A - Pedigree of FRPA019; **B** - RNA studies from FRPA019 with RT-PCR product demonstrating wild type and truncated allele in RPA019 and RPA020. Sanger sequencing of RT-PCR product demonstrates deletion of 36 amino acids of exon 10 in RPA019 and RPA020. **C** - PKD1 variant generates new cryptic donor site and results in in-frame deletion of part of exon 10. **D** - pedigree F19F00138; **E** - Visualisation of BAM files from short read genome sequencing data of 19F00138 demonstrating 19bp intronic deletion; **F** - RNA studies demonstrating retention of intron 31 in RBW401.

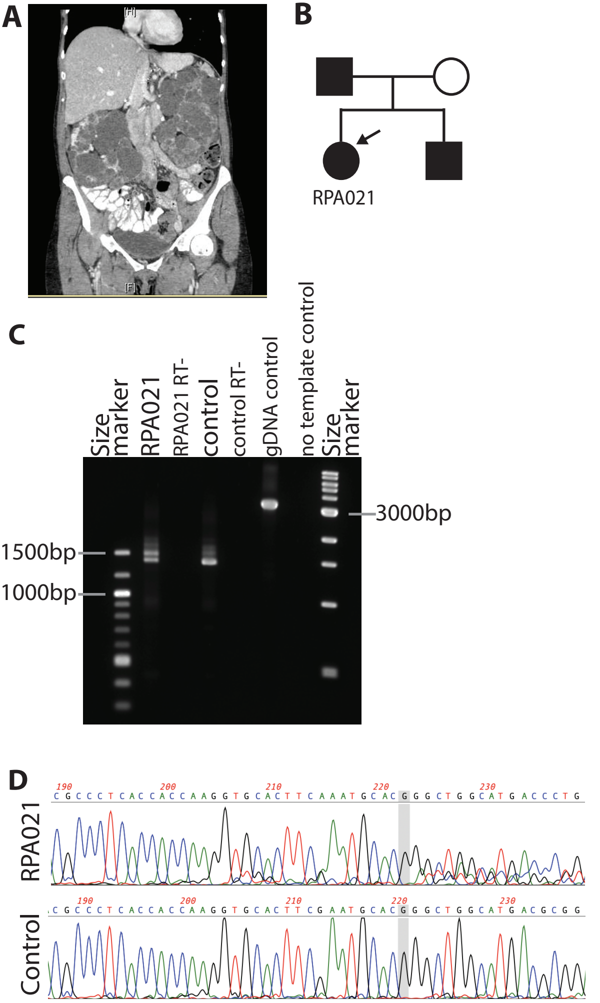

Supplementary Figure 2: Splicing studies in FRPA021:
A - Coronal section from Abdominal CT imaging showing massively enlarged native kidneys. B - Pedigree; C - RT-PCR studies; D - Sanger sequencing of RT-PCR product showing retention of *PKD1* intron 18 in one allele in RPA021.

Supplementary Figure 3: RG_044 Pedigree

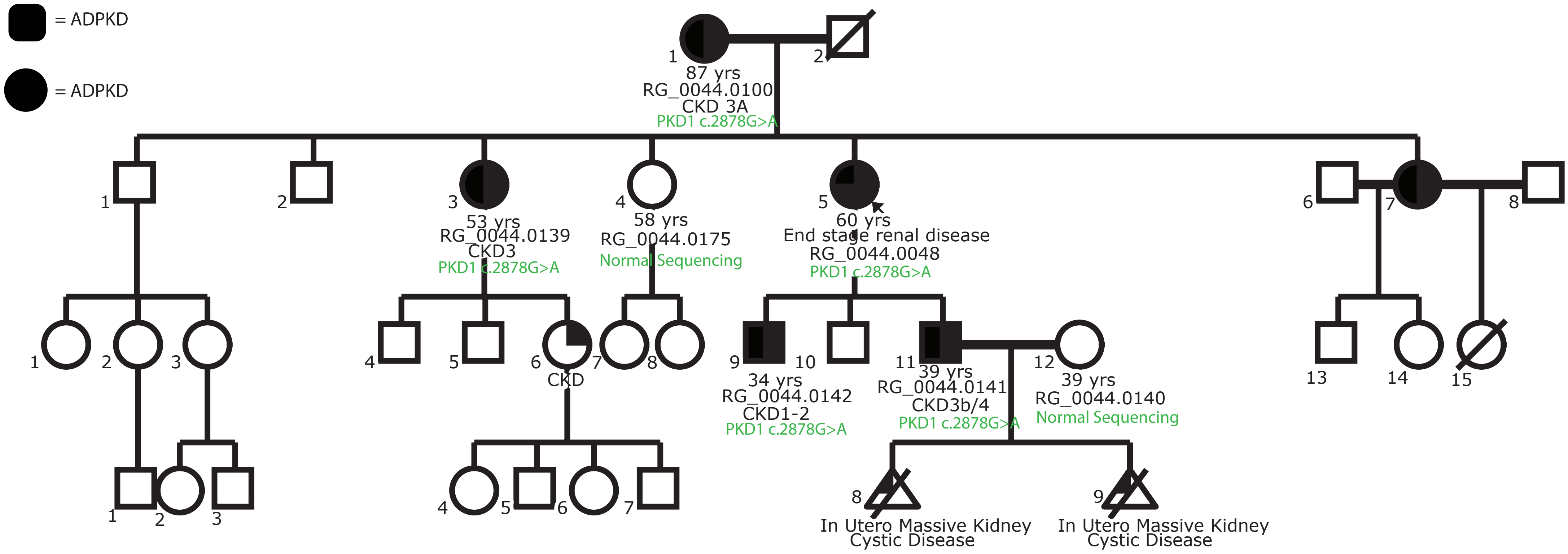

Supplementary Figure 3: RG_044 Pedigree. Sequencing results highlighted in green. Shows PKD1 c.2878G>A variant segregates with phenotype in multiple family members.

Supplementary Table 1: Primer Sequences and PCR Conditions

Region Covered	Primer Label	Direction	Primer Sequence 5' - 3'	PCR Enzymes and Conditions	Samples
PKD1 exon 37 - 39	PKD1_RTPCR_37FOR	Forward	gtcttgctggaagccctgtac	Amplitaq 360 DNA Pol + 0.5M Betaine and 5% DMSO<br>94°C 3 min; then 35 cycles of 95°C 30 sec, 62°C 30 sec, 72°C 30 sec; final extension of 72°C for 5 min. 1.8% Agarose gel.	RBW403, RPA028, RPA014
	PKD1_RTPCR_39REV	Reverse	agctctgggctggactggtt		
PKD1 exon 5 - 12	PKD1_RTPCR_5F	Forward	ggggccccacggacctctgg	Long Amp Taq Pol + 0.5M Betaine and 5% DMSO<br>Touch-down protocol of initial step 95°C 2 min; followed by 14 cycles 98°C 20 sec, 69°C 15 sec with decreasing 0.5°C per cycle, 72°C 4 min; then followed by 25 cycles 98°C 20 sec, 62°C 15 sec, 72°C 4 min; final extension 72°C 5 min. Modified from Tan et al 2014^. 1.5% Agarose gel.	RPA019
	TANLR2(2_12)R^	Reverse	ccacggttacgttgtagttcacggtgacg		
PKD1 exon 31 - 34	Mut4F	Forward	ggactcgtccgtgctggac	Amplitaq 360 DNA Pol + 5% DMSO<br>94°C 3 min; then 35 cycles of 95°C 30 sec, 58°C 30 sec, 72°C 30 sec; final extension of 72°C for 5 min. 1.5% Agarose gel.	RBW401
	AH3B2	Reverse	tccatgtgggtgtcttgggtaggg		
PKD1 exon 15 - 21	ROSLR4(15_21)F#	Forward	agcgcaactacttggaggccc	Long Amp Taq Pol + 0.5M Betaine and 5% DMSO<br>94°C 1min; then 35 cycles of 94°C 30 sec, 63°C 30 sec, 67°C 3 min; final extension 65°C 7 min. 1.8% Agarose gel.	RPA021
	PKD1_21RTPCR_REV	Reverse	ctgccgctcgtgcttgggct		
PKD1 exon 18 -19	PKD1_18RTPCR_FOR	Forward	caacaagacgctggtgctgg		RPA021*
	PKD1_19RTPCR_REV	Reverse	tgcccttgtagacacagaac		

*used for Sanger sequencing

^ Tan AY, Michaeel A, Liu G et al: Molecular diagnosis of autosomal dominant polycystic kidney disease using next-generation sequencing. J Mol Diagn 2014; 16: 216–228.

#Rossetti S, Consugar MB, Chapman AB et al: Comprehensive molecular diagnostics in autosomal dominant polycystic kidney disease. J Am Soc Nephrol 2007; 18: 2143–2160.

Supplementary Table 2: Patient Characteristics and Variant Classification

Family ID	Patient ID	Sex	CKD stage	eGFR/Age (years)	Kidney Imaging	Extra-renal features	Family History of ADPKD	Previous Sequencing	Testing Motivation	Variant idenfitifed in this study	Variant Classification	ACMG classification criteria
FRBW403	RBW403	M	1	>90/31	R kidney length 14.4cm, L kidney length 16.2cm; multiple bilateral simple renal cysts	multiple hepatic cysts; No hx ICA	Affected father (ESKD 44yo)	LR-PCR PKD1 and PKD2 and MPS of PCR product (variant reported likely benign); Diagnostic GS	To facilitate cascade-testing in wider family	PKD1:c.11017-25A>G	Likely Pathogenic	This variant is absent from population databases (PM2). RNA studies demonstrate that exon skipping and partial retention of IVS37 occur; both outcomes are predicted to cause loss of function (PS3_strong).
FRPA028	RPA028	F	1	>90/23	R kidney length 11.9cm; L kidney length 11.9cm; multiple kidney cysts bilaterally;	no hepatic cysts; No hx ICA	Father affected; paternal grandmother affected (dialysis at 47yo)	Diagnostic GS (variant reported as VUS)	To facilitate cascade-testing in wider family	PKD1:c.11017-25A>C	Likely Pathogenic	This variant is absent from population databases (PM2). RNA studies demonstrate that exon skipping and partial retention of IVS37 occur; both outcomes are predicted to cause loss of function (PS3_strong).
FRPA014	RPA014	F	5T	ESKD/56	bilateral, enlarged, cystic kidneys; L kidney length 15cm; R kidney length 14.8cm	diffuse liver cysts; No hx ICA	Affected brothers, mother , maternal aunt and maternal grandmother	Diagnostic GS	To facilitate cascade-testing in young-adult child	PKD1:c.11017-10C>A	Likely Pathogenic	This variant is absent from population databases (PM2). RNA studies demonstrate that exon skipping and partial retention of IVS37 occur; both outcomes are predicted to cause loss of function (PS3_strong). This variant has been previously reported as pathogenic in the literature (PP5).
FRPA019	RPA019	F	1	>90/35	R kidney 12.6cm: L kidney 12.7cm; multiple cysts bilaterally	no hepatic cysts; No hx ICA	Affected mother and brother	LR-PCR PKD1 and PKD2 and MPS of PCR product (variant reported as VUS)	To inform family planning	PKD1:c.1991C>T	Likely Pathogenic	This variant is absent from population databases (PM2). RNA studies demonstrated that this variant alters splicing, causing an in-frame deletion of 36 amino acid in the extracellular region of PC1. This deletion will disrupt the LDL domain, along with glycosylation sites and disulfide bonds within the region (PS3_strong). This variant segregated in multiple affected family members (PP1).
	RPA020	F	3b	44/69	numerous cysts both kidneys	not reported						
F19F00138	RBW401	F	1	>90/30	R kidney length 12.1cm, L kidney length 11.9cm; multiple bilateral simple renal cysts	not reported	Affected maternal grandmother. Affected mother and maternal uncle with ESKD		To inform family planning - for IVF and PGD	PKD1:c.10167+25_10167+43del	Likely Pathogenic	RNA studies demonstrated retention of intron 31, creating a frameshifting insertion and loss of function (PS3_strong). This variant segregated in multiple affected family members (PP1). It has been reported previously in multiple affected individuals in the literature (PP5). It is present at a low frequency in population databases not greater than what would be expected given the known disease incidence.
	19F00138	F	1	>90/25	R kidney length 13.2cm, L kidney length 11.7cm; multiple bilateral simple renal cysts	not reported		Diagnostic GS				
FRPA021	RPA021	F	5T	ESKD/52	bilateral, enlarged, cystic kidneys	massive liver cysts requring liver transplant at 48yo; No hx ICA	Affected Father and brother (ESKD 47yo)	Diagnostic GS (variant reported as VUS)	To facilitate cascade-testing in young-adult children	PKD1:c.7489+5G>A	Likely Pathogenic	This variant is absent from population databases (PM2). RNA studies demonstrated this variant resulted in retention of 93 base pairs of intron 18 introducing a premature stop codon (PS3_strong).

RG_0044	RG_0044.0048	F	5	ESKD/58	bilateral, enlarged, cystic kidneys	not reported	see Supp. Fig 2	LR-PCR PKD1 and PKD2 and MPS of PCR prodcut	To facilitate cascade-testing in wider family	PKD1:c.2878G>A	Likely Pathogenic	This variant segregated in six affected family members, across three generations (PP1_Moderate). It has been previously reported as likely pathogenic in the literature, and identified in multiple affected, unrelated individuals (PMID: 33168999, 23300259, 31740684). It is present at a low frequency in population databases not greater than what would be expected given the known disease incidence. (PS4_Moderate). In silico tools (REVEL, 0.865, and CADD, 24.6) predict this variant as pathogenic (PP3). A missense change impacting the same residue (p.Gly960Asp) has been previously reported as pathogenic (PMID: 22383692) (PM5_supporting)
FRPA017	RPA017	F	3b	41/38	R kidney length 18cm; L kidney length 20cm; numerous bilateral simple renal cysts	not reported; No hx ICA	Two affected maternal uncles with stage 3 CKD; maternal grandmother ESKD 81yo	Diagnostic GS	To inform family planning - for IVF and PGD	PKD1:c.10118C>A	Pathogenic	This variant is absent from population databases (PM2). It is predicted to cause loss of function (PVS1). This variant was confirmed de novo in the proband (PS2).
	RPA015	F	3a	50/71	R kidney length 10.1cm; L kidney length 14.9cm; multiple bilateral simple renal cysts	not reported; No hx ICA				PKD2:c.1249C>T	Pathogenic	This variant is absent from population databases (PM2). It is predicted to cause loss of function (PVS1). It is a well-established pathogenic variant and has been reported as multiple times in the literature (PP5)
FRPA007	RPA007	F	5T	ESKD/45	massively enlarged kidneys bilaterally with numerous cysts	massively enlarged liver, required liver/kidney translplant; No hx ICA	Affected mother	Diagnostic GS (variant reported as VUS)	To facilitate cascade-testing in young-adult children	PKD1:c.8471A>G	VUS	This variant is absent from population databases (PM2).

CKD = Chronic Kidney Disease; ESKD = End Stage Kidney Disease; ICA = Intracranial Aneurysm; GS = Genome Sequencing; VUS = Variant of Uncertain Significance; MPS = Massivelly Parallel Sequencing; LR-PCR = Long Range PCR; IVF = In vitro fertilization; PGD = Pre-implantation genetic diagnosis
